# Sociodemographic Determinants of Smoking Behavior Among Adults in the Eastern Province of Saudi Arabia: A Cross-Sectional Study

**DOI:** 10.7759/cureus.95995

**Published:** 2025-11-03

**Authors:** Haidar Alabdrabulridha, Ryhana M Aljumaiah, Wedad Nasser Alturifi, Amani Nasser Alturifi, Abdullah Almethen

**Affiliations:** 1 Preventive Medicine Department, King Faisal General Hospital, Hofuf, SAU; 2 Internal Medicine Department, King Fahad General Hospital, Hofuf, SAU; 3 Preventive Medicine Department, King Abdulaziz National Guard Hospital, Al Ahsa, SAU; 4 Nursing Department, King Abdulaziz National Guard Hospital, Al Ahsa, SAU; 5 Patient Services Department, King Faisal General Hospital, Hofuf, SAU

**Keywords:** cross-sectional study, public health, saudi arabia, smoking behavior, socio-demographic determinants

## Abstract

Background

Smoking remains a leading cause of preventable morbidity and mortality worldwide. This study aims to determine the sociodemographic factors associated with smoking behavior among adults in the Eastern Province of Saudi Arabia.

Methods

A cross-sectional survey of 551 adults aged 15-65 years was conducted between December 2024 and February 2025. Data were collected via a validated self-administered questionnaire distributed through WhatsApp (Meta Platforms, Inc., Menlo Park, CA). Associations between smoking status and sociodemographic variables (age, gender, education, income, and occupation) were analyzed using chi-square tests with a significance level of p < 0.05.

Results

Of 551 participants, 165 (29.9%) were current smokers, 328 (59.5%) non-smokers, and 58 (10.5%) passive smokers. Smoking prevalence was significantly higher among males (154, 37.8%) than among females (11, 7.6%) (p = 0.001). Participants with a diploma (45, 37.5%) had the highest smoking rate, while those with postgraduate degrees (nine, 17.6%) had the lowest (p = 0.003). Smoking was also associated with overweight status (65, 37.8%) and private-sector employment (63, 50.8%) (p < 0.05).

Conclusion

Smoking prevalence remains high among adults in the Eastern Province, particularly among men, those with lower educational levels, and private-sector workers. Educational attainment may act as a protective factor. These findings support the implementation of targeted public health strategies such as workplace-based smoking cessation programs, awareness campaigns focusing on low-education groups, and stricter enforcement of tobacco control policies to reduce smoking prevalence in high-risk populations.

## Introduction

Smoking remains a major global public health concern and one of the leading preventable causes of morbidity and mortality. According to the World Health Organization (WHO), tobacco use is the second most significant risk factor for death worldwide and was labeled a global epidemic in 2019 [1]. In Saudi Arabia, smoking rates have stayed high, and the country was once ranked fourth in the world for tobacco imports [2].

There is a clear difference in smoking rates between men and women in Saudi Arabia, and about 32.3% of men smoke, compared to 13.5% of women [3]. Moreover, adolescence is a critical period when individuals are more likely to experiment with smoking. According to WHO data, youth tobacco use remains a concern in Saudi Arabia, with national surveys indicating that a significant proportion of students have experimented with smoking before the age of 15 [4]. Starting this habit early makes it much more likely that someone will keep using tobacco as they get older.

Socioeconomic factors also play a significant role. Higher income levels have been associated with increased tobacco use, likely due to greater purchasing capability [5]. The previously low cost of tobacco and limited taxation in Saudi Arabia made it widely accessible, especially among youth. Although recent tax increases have aimed to reduce consumption, studies indicate that smoking is still more prevalent among individuals with higher socioeconomic status [6,7].

Meanwhile, education seems to have the opposite effect, as individuals with higher education levels are less likely to smoke compared to those with lower education levels [3]. Therefore, this study aims to determine the sociodemographic factors associated with smoking behavior among adults in the Eastern Province of Saudi Arabia.

## Materials and methods

Study design and setting

A cross-sectional study was conducted to assess the association between smoking behavior and sociodemographic characteristics among adults in the Eastern Province of Saudi Arabia. Data were collected over a three-month period, from December 2024 to February 2025.

Study population and sampling

The inclusion criteria were as follows: (a) participants aged 15 years or older who were able to provide informed consent and complete the questionnaire in either Arabic or English; (b) residents of the province at the time of data collection; and (c) both smokers (cigarette, shisha, or e-cigarette users) and non-smokers, to allow comparison between groups. Participants were excluded if they were younger than 15 years, not residents of the province, had severe cognitive impairments, or were unable to understand the survey language.

The minimum required sample size was calculated using the standard prevalence formula $n = (Z^2 &times; p &times; (1 - p)) / d^2$. Assuming a 95% confidence level (Z = 1.96), a prevalence of smoking (P) of 0.5 to ensure maximum variability, and a margin of error (E) of 0.05, the required sample size was 384 participants. To compensate for potential nonresponse or incomplete data, a total of 500 adults were ultimately required. Participants were selected through a non-random convenience sampling approach, with efforts made to include both genders and a range of ages, education levels, and employment statuses.

Data collection

Data were collected using a self-administered, structured online questionnaire distributed via WhatsApp (Meta Platforms, Inc., Menlo Park, CA) and social media networks. Participants were encouraged to share the link with peers and relatives to expand the reach of the survey. The questionnaire was developed based on previously established instruments used in smoking-related research and reviewed by public health specialists to ensure content validity and clarity.

The questionnaire consisted of 44 items organized into five main sections. The first section collected sociodemographic data, including age, gender, marital status, education, employment type, and income. The second section covered health-related factors, such as body mass index (BMI), presence of chronic diseases, and level of physical activity. The third section assessed smoking behavior, including smoking status (current, former, non-smoker, or passive), the type of product used (cigarette, shisha, or e-cigarette), frequency of use, and age of initiation. The fourth section explored attitudes and beliefs toward smoking, focusing on awareness of health risks, social acceptability, and perception of smoking bans. The final section addressed smoking cessation experiences, including previous quit attempts, clinic visits, and barriers to quitting.

Ethical considerations

Ethical approval was obtained from the Institutional Review Board (IRB) at King Saud University, Riyadh, Saudi Arabia (Approval No. KSU-HE-24-1087). Participants were informed about the objectives of the study, and electronic informed consent was obtained before participation. Participation was voluntary, and respondents were assured of the confidentiality and anonymity of their responses. No personal identifiers were collected. All responses were securely stored in a password-protected database accessible only to the research team to ensure data security and participant confidentiality. 

Data analysis

All data were analyzed using IBM SPSS Statistics version 27 (IBM Corp., Armonk, NY). Descriptive statistics, including frequencies and percentages, were used to summarize sociodemographic characteristics, smoking status, and smoking-related behaviors. The Pearson chi-square test was applied to examine associations between smoking status (current smoker, non-smoker, and passive smoker) and sociodemographic variables, including age, gender, education level, marital status, BMI, employment sector, and field of work. When necessary, the Exact Probability Test was used to confirm the results. A p-value of less than 0.05 was considered statistically significant.

## Results

A total of 551 adults participated in the study conducted in the Eastern Province of Saudi Arabia. After data screening, all responses that met the inclusion criteria were retained for analysis, while participants who provided incomplete or inconsistent information were excluded.

As shown in Table 1, almost half of the participants were aged 30-44 years (270, 49.0%), while 175 (31.8%) were aged 15-29 years. The majority were male (407, 73.9%) and married (397, 72.1%). More than half held a bachelor’s degree (278, 50.5%), and approximately 398 (72.2%) were employed, most of whom worked in governmental sectors (241, 61.0%).

**Table 1:** Socio-demographic and health-related characteristics of the study participants (N = 551)

Bio-demographics		No	%
Age in years	15-29	175	31.8%
	30-44	270	49.0%
	45-60	100	18.1%
	60-65	6	1.1%
Gender	Male	407	73.9%
	Female	144	26.1%
Qualification	Secondary education	102	18.5%
	Diploma	120	21.8%
	Bachelor degree	278	50.5%
	Post-graduate	51	9.3%
Marital status	Single	138	25.0%
	Married	397	72.1%
	Divorced/widow	16	2.9%
Are you pregnant	Yes	8	1.9%
	No	405	98.1%
Body mass index	Underweight	31	5.6%
	Normal weight	199	36.1%
	Overweight	172	31.2%
	Obese	149	27.0%
Family size	Not living alone	14	2.5%
	2 persons	72	13.1%
	3-4 persons	221	40.1%
	> 5 persons	244	44.3%
Employment	Unemployed	80	14.5%
	Student	73	13.2%
	Employed‎	398	72.2%
Nature of work	Governmental	241	61.0%
	Semi-governmental	24	6.1%
	Private	124	31.4%
	Free work	6	1.5%
Field of work	Health care filed	198	35.9%
	Non-health care field	353	64.1%
Monthly income	< 5000 SR	129	23.4%
	5000-10000 SR	132	24.0%
	10000-15000 SR	172	31.2%
	> 15000 SR	118	21.4%
Chronic diseases	None	442	80.2%
	DM	29	5.3%
	HTN	58	10.5%
	Others	12	2.2%
	Cardiovascular disease	7	1.3%
	Asthma	3	0.5%
Practice sports	Yes	319	57.9%
	No	232	42.1%
Severity of practiced sports	Light sports	143	44.8%
	Intermediate sports	151	47.3%
	Heavy sports	25	7.8%
Times to exercise per week	1-2 times	156	44.4%
	3-4 times	138	39.3%
	5-7 times	57	16.2%

Regarding lifestyle and health characteristics, 319 (57.9%) of participants reported practicing sports, and 199 (36.1%) had a normal BMI. The most common chronic conditions were hypertension (58, 10.5%) and diabetes mellitus (29, 5.3%) (Table 1).

Table 2 presents the associations between participants’ sociodemographic characteristics and smoking status. Significant associations were observed for gender, educational qualification, BMI, employment status, nature of work, and field of work (p < 0.05).

**Table 2:** Factors associated with smoking among study adolescents, Eastern Providence * indicates statistically significant results; ^ indicates results that are not statistically significant.

Factors		Smoking status						p-value
		Current smoker		Non-smoker		Passive-smoker		
		No	%	No	%	No	%	
Age in years	15-29	53	30.3%	108	61.7%	14	8.0%	0.079^
	30-44	83	30.7%	148	54.8%	39	14.4%	
	45-60	27	27.0%	68	68.0%	5	5.0%	
	> 60	2	3.33%	4	66.7%	0	0.0%	
Gender	Male	154	37.8%	208	51.1%	45	11.1%	0.001*
	Female	11	7.6%	120	83.3%	13	9.0%	
Qualification	Secondary education	30	29.4%	69	67.6%	3	2.9%	0.003*
	Diploma	45	37.5%	67	55.8%	8	6.7%	
	Bachelor degree	81	29.1%	160	57.6%	37	13.3%	
	Post-graduate	9	17.6%	32	62.7%	10	19.6%	
Marital status	Single	46	33.3%	83	60.1%	9	6.5%	0.164
	Married	115	29.0%	233	58.7%	49	12.3%	
	Divorced/widow	4	25.0%	12	75.0%	0	0.0%	
Are you pregnant	Yes	2	25.0%	5	62.5%	1	12.5%	0.971^
	No	117	28.9%	240	59.3%	48	11.9%	
Body mass index	Underweight	8	25.8%	19	61.3%	4	12.9%	0.049*
	Normal weight	47	23.6%	126	63.3%	26	13.1%	
	Overweight	65	37.8%	97	56.4%	10	5.8%	
	Obese	45	30.2%	86	57.7%	18	12.1%	
Family size	Not living alone	4	28.6%	9	64.3%	1	7.1%	0.402
	2 persons	18	25.0%	47	65.3%	7	9.7%	
	3-4 persons	75	33.9%	118	53.4%	28	12.7%	
	> 5 persons	68	27.9%	154	63.1%	22	9.0%	
Employment	Unemployed	11	13.8%	64	80.0%	5	6.3%	0.001*
	Student	17	23.3%	49	67.1%	7	9.6%	
	Employed‎	137	34.4%	215	54.0%	46	11.6%	
Nature of work	Governmental	63	26.1%	148	61.4%	30	12.4%	0.001*^
	Semi-governmental	6	25.0%	12	50.0%	6	25.0%	
	Private	63	50.8%	52	41.9%	9	7.3%	
	Free work	3	50.0%	2	33.3%	1	16.7%	
Field of work	Health care filed	51	25.8%	115	58.1%	32	16.2%	0.004*
	Non-health care field	114	32.3%	213	60.3%	26	7.4%	
Monthly income	< 5000 SR	34	26.4%	85	65.9%	10	7.8%	0.383
	5000-10000 SR	46	34.8%	75	56.8%	11	8.3%	
	10000-15000 SR	52	30.2%	100	58.1%	20	11.6%	
	> 15000 SR	33	28.0%	68	57.6%	17	14.4%	
Chronic diseases	Yes	32	29.4%	68	62.4%	9	8.3%	0.648
	No	133	30.1%	260	58.8%	49	11.1%	
Practice sports	Yes	100	31.3%	190	59.6%	29	9.1%	0.371
	No	65	28.0%	138	59.5%	29	12.5%	
Times to exercise per week	1-2 times	50	32.1%	94	60.3%	12	7.7%	0.074
	3-4 times	41	29.7%	78	56.5%	19	13.8%	
	5-7 times	16	28.1%	40	70.2%	1	1.8%	

Males (154, 37.8%) were more likely to be current smokers than females (11, 7.6%) (p = 0.001). Smoking prevalence was also higher among those with a diploma (45, 37.5%) compared to those with postgraduate degrees (nine, 17.6%) (p = 0.003).

Regarding employment, employed participants (137, 34.4%) reported a higher rate of smoking than unemployed individuals (11, 13.8%) (p = 0.001). Similarly, participants working in private sectors (63, 50.8%) and non-healthcare fields (114, 32.3%) were more likely to smoke compared to those in governmental or healthcare positions (p = 0.001 and p = 0.004, respectively).

A significant association was also found between BMI and smoking status (p = 0.049), with overweight individuals (65, 37.8%) showing a higher proportion of smokers. No statistically significant differences were observed regarding age, marital status, family size, income, chronic diseases, or physical activity (p > 0.05).

**Figure 1:**
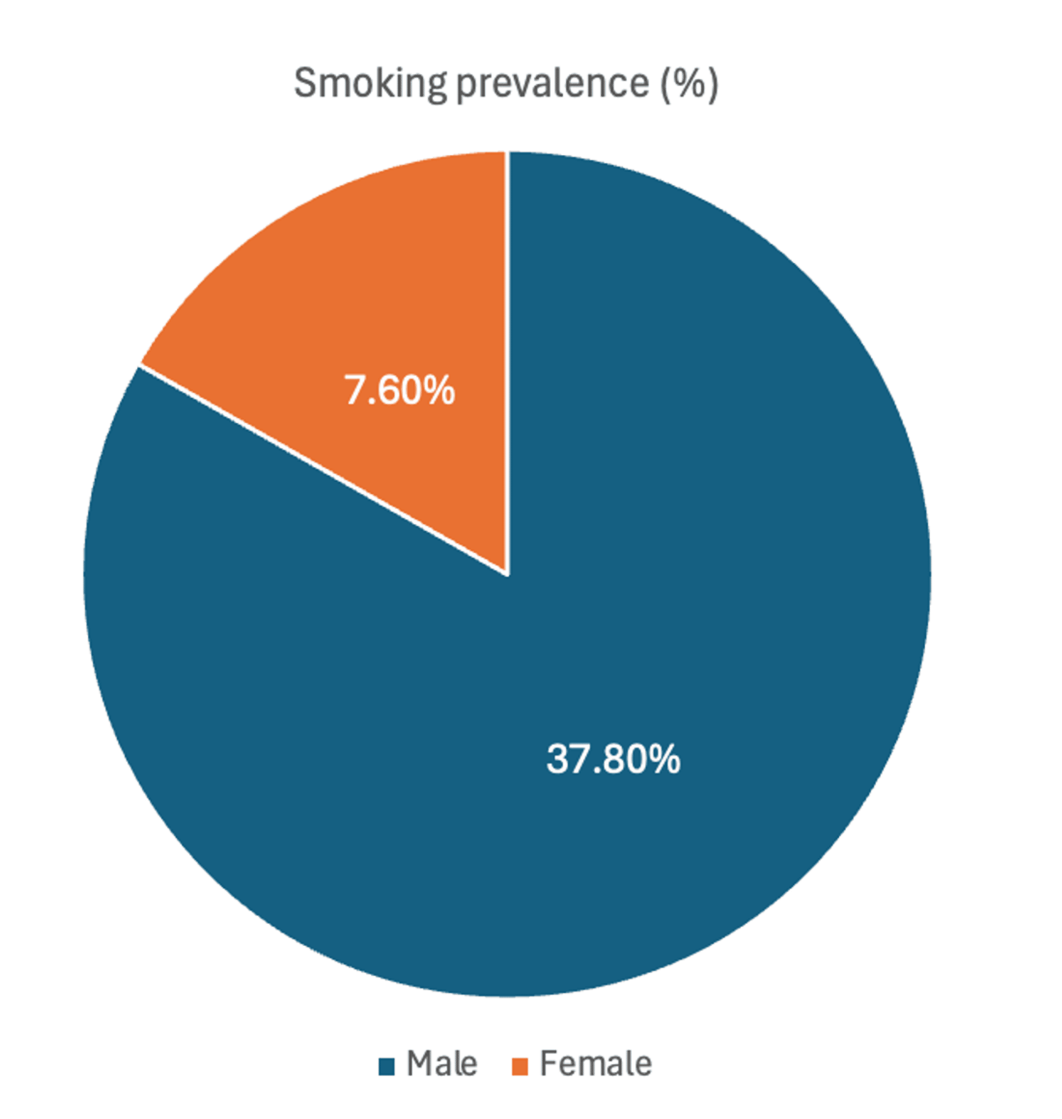
Smoking prevalence by gender among adults in the Eastern Province, Saudi Arabia

Figure 1 illustrates smoking prevalence by gender among adults in the Eastern Province. The prevalence of current smoking was significantly higher among males (154, 37.8%) compared with females (11, 7.6%) (p = 0.001).

## Discussion

This study examined the association between smoking behavior and sociodemographic factors among adults in the Eastern Province of Saudi Arabia. The prevalence of current smoking in this study was 165 (29.9%), higher than the 2020 WHO global average of 22.3% but consistent with national estimates of 20-30% reported in previous Saudi studies [8,9]. This finding highlights that tobacco use remains a persistent public health issue in Saudi Arabia, particularly in the Eastern Province.

The majority of participants were within the 30-44 age group, predominantly male, and held bachelor's degrees. Smoking prevalence was notably higher among males, reflecting social and cultural acceptance of smoking among men in Saudi society, as reported in previous regional research [3,10]. The association between gender and smoking remains strong across the Gulf region, emphasizing the need for gender-specific prevention and cessation strategies.

Education also showed a clear association with smoking behavior. Although participants with higher education levels were expected to have greater awareness of smoking-related risks, smoking prevalence was not substantially lower in this group. This suggests that awareness alone may not lead to behavioral change without supportive interventions or environmental modifications [6].

Marital status was another significant determinant. Married individuals were less likely to smoke compared with single participants, consistent with evidence indicating that family responsibilities and spousal influence may reduce the likelihood of smoking initiation or encourage cessation [11,12]. Employment status also influenced smoking behavior, with higher rates observed among individuals working in governmental sectors. This may be related to occupational stress, social norms in workplaces, or the accessibility of smoking opportunities during breaks [13].

Regarding health-related demographics, overweight and obesity were prevalent among participants, as were chronic conditions such as hypertension and diabetes. Although these conditions were not directly linked to smoking status in this sample, their presence underscores the broader health burden associated with tobacco use [14].

Sociocultural influences were strongly linked to smoking initiation and continuation. Among participants, 73 (13.3%) reported receiving their first cigarette from friends or college peers, highlighting the crucial role of close social circles in shaping smoking behavior [11,15]. Moreover, the presence of smokers within households (reported by 393 (71.4%) of participants) reinforces the influence of family environment and social modeling on smoking habits [16]. The high use of shisha and e-cigarettes (190, 34.5%) reflects emerging trends among younger adults and the misconception that these alternatives are less harmful than traditional cigarettes [3,6,17]. This highlights the need to address inaccurate health risk perceptions through targeted educational and behavioral interventions aimed at correcting these beliefs and reducing tobacco uptake among youth.

Finally, while many smokers had attempted to quit, the low utilization of cessation clinics points to gaps in public health infrastructure and accessibility. Among those who reported previous quit attempts, common barriers included lack of awareness about available cessation services, limited accessibility, and the perception that quitting could be achieved without professional support. These findings underscore the need to strengthen public health initiatives that promote awareness and accessibility of evidence-based cessation programs. Work-related stress and lack of time were identified as barriers to quitting, particularly among employed adults [18]. Developing workplace-based cessation support and promoting stress management strategies could enhance quit success in this population [19].

While this study provides insight into the sociodemographic determinants of smoking behavior in the Eastern Province, several limitations should be considered when interpreting the findings. First, the use of a cross-sectional design restricts the ability to establish causal relationships between sociodemographic factors and smoking behavior. Second, the reliance on self-reported data through an online questionnaire may have introduced recall and social desirability biases, particularly given the cultural stigma associated with smoking among women in Saudi Arabia. Additionally, data were collected using a self-administered online survey distributed via social media platforms, which may have introduced selection bias, as individuals with internet access and interest in health-related topics were more likely to participate. Participants were also encouraged to share the survey link with peers and relatives, introducing an element of snowball sampling. While this facilitated wider participation, it may have resulted in clustering within social networks and limited the representativeness of the sample. Consequently, these factors may restrict the generalizability of the findings to the broader adult population in the Eastern Province. Finally, important variables such as psychological stress, peer influence, and detailed smoking patterns (e.g., frequency, duration, nicotine dependence) were not fully explored and should be addressed in future research.

## Conclusions

In conclusion, the present study highlights the influence of sociodemographic and social factors on smoking behavior among adults in the Eastern Province of Saudi Arabia. The findings revealed that gender, marital status, education level, and employment type were significantly associated with smoking, with higher prevalence observed among men, diploma holders, and private-sector employees. Educational attainment appeared to play a protective role, while family environment and social context remained strong determinants of smoking initiation and continuation.

The study emphasizes the need for culturally appropriate prevention and cessation strategies that target specific high-risk groups and address social influences related to smoking. Community-based awareness campaigns, family-centered programs, and workplace cessation support are recommended to reduce smoking prevalence and promote healthy behavior among adults in the region.
